# The association of serum betaine concentrations with the risk of new-onset cancers: results from two independent nested case-control studies

**DOI:** 10.1186/s12986-023-00755-y

**Published:** 2023-10-30

**Authors:** Hailun Xie, Kangping Zhang, Yaping Wei, Guotian Ruan, Heyang Zhang, Shuqun Li, Yun Song, Ping Chen, Lishun Liu, Binyan Wang, Hanping Shi

**Affiliations:** 1 Department of Gastrointestinal Surgery/Clinical Nutrition, Beijing International Science and Technology Cooperation Base for Cancer Metabolism and Nutrition, Key Laboratory of Cancer FSMP for State Market Regulation, Capital Medical University Affiliated Beijing Shijitan Hospital, Beijing, 100038 China; 2 Department of Neurobiology, School of Basic Medical Sciences, Capital Medical University, Beijing, 100069 China; 3 Key Laboratory of Precision Nutrition and Food Quality, Ministry of Education, Department of Nutrition and Health, College of Food Sciences and nutritional engineering, China Agricultural University, Beijing, 100083 China; 4 Shenzhen Evergreen Medical Institute, Shenzhen, China

**Keywords:** Betaine, Incident cancer, Hypertension, Risk

## Abstract

Evidence from epidemiologic studies on the association of circulating betaine levels with the incident risk of cancer has been inconsistent. We aimed to investigate the prospective association of serum betaine concentrations with the risk of cancer. We performed two, nested, case-control studies utilizing data from the “H-type Hypertension Prevention and Control Public Service Project” (HHPCP) and the China Stroke Primary Prevention Trial (CSPPT), with 2782 participants (1391 cancer cases and 1391 matched controls) in the discovery cohort, and 228 participants (114 cancer cases and 114 matched controls) in the validation cohort. Odds ratios (OR) of the association between betaine and cancer were calculated using conditional logistic regression models. There was an association between serum betaine as a continuous variable and total cancer (OR = 1.03, 95%CI = 0.99–1.07, p = 0.097). Among cancer subtypes, a positive association was found between serum betaine and the risk of lung cancer, and an inverse association was found with other cancers. Interestingly, a U-shaped association was observed between serum betaine and digestive cancers, with a turning point of 5.01 mmol/L for betaine (betaine < 5.01 mmol/L, OR = 0.82, 95%CI = 0.59–1.14, p = 0.228; betaine ≥ 5.01 mmol/L, OR = 1.08, 95%CI = 1.01–1.17, p = 0.036). In the validation cohort, a significant association between serum betaine as a continuous variable and total cancer (OR = 1.48, 95%CI = 1.06–2.05, P = 0.020) was also found. High serum betaine was associated with increased risk of total cancer and lung cancer, and a U-shaped association was found with the risk of digestive cancers, with a turning point at about 5.01 mmol/L.

**Supplementary Information:**

The online version contains supplementary material available at 10.1186/s12986-023-00755-y.

## Novelty & impact statements

In this nested, case-control study, we found that high serum betaine was associated with an increased risk of cancer. In terms of tumor types, serum betaine had a positive correlation with lung cancer, a U-shaped correlation with gastrointestinal cancer, and a negative correlation with other tumors. These analyses may have potential scientific and clinical implications for cancer prevention and dietary guidance.

## Introduction

Cancer is the first or second leading cause of premature death in more than half of the countries around the world [1]. It is estimated that about 19.3 million new cancer cases occurred globally in 2020, with China leading the world in the number of new cancer cases (about 4.57 million). That’s an average of about 125,000 people diagnosed with cancer every day in China, or, 8.7 people diagnosed with cancer every minute, which indicates a very high burden of cancer [2]. Although much attention has been given to cancer prevention efforts, new cancer prevention strategies that are supplemented by up-to-date scientific evidence, are still needed.

Cancer cells require one-carbon units in order to support their biosynthesis, proliferation, and survival. As an important methyl donor in one-carbon metabolism, betaine plays a crucial role in the occurrence of many chronic diseases, including cancer [3–5]. Betaine, a kind of human nutrient, can be obtained from dietary intake of foods [6]. In addition to dietary sources, betaine is synthesized from the two-step oxidation reaction of choline through mitochondria choline dehydrogenase and betaine aldehyde dehydrogenase, endogenously [4]. However, the relationship between betaine and cancer is still controversial. Some studies have shown that low levels of betaine can increase the likelihood of DNA damage and genetic mutations caused by abnormal methylation, which may be associated with carcinogenesis [7, 8]. Many studies have found that an increase in serum betaine is related to a reduced risk of breast, colorectal, and pancreatic cancers [9–11]. A meta-analysis by Sun et al. [12] showed that increased intake of betaine is associated with reduced cancer incidence. The results of a 2019 meta-analysis also showed that both dietary intake and serum betaine levels are inversely associated with cancer incidence [13]. However, betaine metabolism in the body is a complex multi-path process. Some studies have found that the downstream transformants of betaine (such as trimethylamine oxide) are associated with an increased risk of cancer. Previous studies have shown that a Western diet increases the risk of developing cancer. Under the action of the gut microbiota, these products are converted into betaine compounds, choline, and carnitine, and metabolized in the liver to carcinogens such as trimethylamine N-oxide (TMAO) [14–16]. Elevations of circulating TMAO have been reported to be related to an increased risk of some cancers [17, 18]. A nested, case-control study found that participants with higher baseline serum levels of betaine showed an increased odds of developing lethal prostate cancer [19]. In addition, alterations in gut microbe-dependent metabolites, which may appear early in disease development, are associated with a higher risk of colorectal cancer [20]. Previous studies have also found that the incidence of cancer in patients with high serum betaine is on the rise [21–23]. Therefore, overdietary intake or endogenous oversynthesis resulting in serum betaine build-up may lead to an increased risk of tumor development.

Given that there are still conflicting views on the relationship between serum betaine and cancer risk, the purpose of this study was to evaluate the prospective relationship between serum betaine concentrations and the subsequent risk of total cancer, digestive cancers, and non-digestive cancers utilizing data from the China H-type Hypertension Prevention and Control Public Service Project (HHPCP) and the China Stroke Primary Prevention Trial (CSPPT) cohorts. Furthermore, we examined possible effect modifiers on the betaine-cancer relationship using a nested, case-control design.

## Methods

### Study population and design

In this nested, case-control study, the discovery cohort was derived from the HHPCP, a community cohort study conducted in Rongcheng, China from 2016 to 2018. The validation cohort came from the CSPPT (clinicaltrials.gov identifier: NCT00794885), a multi-community, randomized, double-blind, controlled trial, conducted in 2 provinces (32 communities) in China from May 19, 2008 to August 24, 2013. The details of the HHPCP cohort and the CSPPT cohort and their study protocols have been described previously [24, 25]. All eligible participants were men and women aged ≥ 35 years with hypertension. Hypertension at the screening and recruitment visit was defined as seated, resting systolic blood pressure (SBP) > 140 mmHg or diastolic blood pressure (DBP) > 90 mmHg, or patients taking antihypertensive medication. The major exclusion criteria included history of physician-diagnosed stroke, myocardial infarction (MI), heart failure, post-coronary revascularization, and/or congenital heart disease. The Ethical Committee of the Institute of Biomedical Sciences of Anhui Medical University approved the two study protocols. All participants or their representative relatives provided written, informed consent. The study was conducted in accordance with the Declaration of Helsinki.

### Population statistics and laboratory assays

Baseline information on demographic, medical history, and lifestyle characteristics was collected through physical examinations, questionnaire interviews, and biological samples. General data included age, sex, family history of cancer, alcohol consumption, smoking, history of diabetes and cancer. Baseline measurements, including height, weight, and body mass index (BMI), were performed by professionally trained individuals, and the mean of multiple measurements was used. Morning serum samples were collected from all participants after a 12-hour overnight fast. Serum betaine was measured by a laboratory using a chemiluminescent immunoassay (New Industrial, Shenzhen, China). Serum betaine was measured for both cohorts at the time the participants were enrolled. Beckman Coulter automatic clinical analyzers were used to measure homocysteine, folate, total cholesterol, triglycerides, high-density lipoprotein cholesterol, and glucose levels in a laboratory. The C677t gene (rs1801133) polymorphisms of 5,10-methylenetetrahydrofolate reductase (MTHFR) were detected with an ABI Prism 7900HT sequence detection system (Life Technologies) using the TaqMan assay.

### Outcome assessment

Cancer incidence was the main outcome in this study, which was derived from the local Centers for Disease Control and Prevention (CDC) surveillance data collected at 2021. Cancer was diagnosed on the basis of positive pathology data. When pathology data were not available, cases were reviewed independently by two physicians. Only when the two physicians reached an agreement, the diagnosis of cancer can be performed. All cancer incidents were reviewed by an independent committee.

### Nested, case-control study

**Fig. 1:**
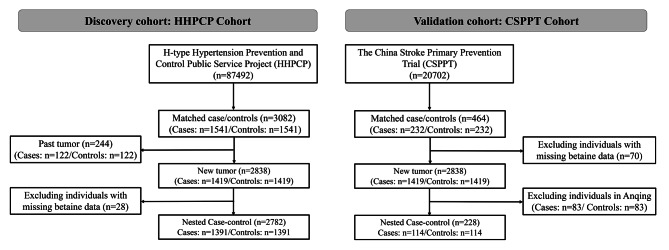
Flow chart of study participants in the nested case-control study within the H-type Hypertension Prevention and Control Public Service Project cohort (2016–2018) and the China Stroke Primary Prevention Trial cohort, (May 2008 to August 2013)

In the HHPCP cohort, cancer occurred in 1541 participants (1.72%). The control group was selected from the study population of individuals who had not developed cancer and whose data were complete. Cases and controls were matched in a 1:1 ratio by age (± 1 year), sex, and region. The original, eligible cohort for this nested, case-control study included 1541 cancer events and 1541 matched controls. After excluding patients with preexisting cancer and those with missing serum betaine data, 2782 participants (1391 cancer cases and 1391 matched controls) were included in the discovery cohort. In the CSPPT cohort, cancer occurred in 232 participants (1.12%). Participants who did not develop cancer during follow-up were selected as controls and were matched in a 1:1 ratio by age (± 1 year), sex, and region. The participants of Anqing region were excluded because they had too many missing values of serum betaine and their numbers were too small. After excluding participants from the Anqing region and those with missing serum betaine data, the final validation cohort included 228 participants (114 new incident cancer cases and 114 matched controls) (Fig. 1).

### Statistical analysis

Continuous variables with a normal distribution were expressed as means ± SD and compared using a generalized paired t-test. Variables with skewed distributions were presented as median ± IQR and compared using the nonparametric Kruskal-Wallis test. Categorical variables were expressed as values (percentages) and compared using chi-square tests. The dose-response association between serum betaine and carcinogenesis was calculated by restricted cubic spline regression (RCS). Odds ratios (ORs) and 95% confidence intervals (CIs) were estimated using conditional logistic regression to assess the association between serum betaine and cancer risk without and with adjustment for age, BMI, sex, smoking status, alcohol consumption, family history of cancer, diabetes, folate, homocysteine, SBP, triglycerides, cholesterol, high-density lipoprotein cholesterol, glucose, and MTHFR C677T. Participants were divided into four subgroups (Q1-Q4) based on quartiles of serum betaine to assess the dose-dependent relationship between serum betaine and cancer risk. In addition, the potential interaction of variables was assessed by a multiplicative model.

All statistical calculations were performed using R software (version 3.3.1; http://www.R-project.org). The statistical test results were considered significant with a two-sided P value < 0.05.

## Results

### Study participants and baseline characteristics

A total of 2782 participants (1391 new incident cancer cases and 1391 matched controls) within the HHPCP cohort and 228 participants (114 new incident cancer cases and 114 matched controls) within the CSPPT cohort were included in this study. In the HHPCP cohort, the mean age for cancer cases was 69.35 (7.78) years, with 776 males (55.8%) and 615 females (44.2%). Among total new cancer cases, 361 (26.0%) were lung cancer, 544 (39.1%) were digestive cancers, and 486 (34.9%) were other cancers. Compared with matched controls, cancer patients were more likely to have higher rates of both former and current smoking (p = 0.011), history of diabetes (p = 0.002), and higher high-density lipoprotein cholesterol. Serum betaine concentration was slightly higher in the cases than in the matched controls, but there was no significant difference (5.80 ug/ml vs. 5.73ug/ml, p = 0.401) (Table 1). In addition, the relationship between serum betaine and cancer risk was also validated in the CSPPT cohort. The mean age for cancer cases in this cohort was 60.86 (7.12) years, with 56 males (49.1%) and 58 females (50.9%). As shown in Table 1, there was a significant difference between baseline serum betaine in the controls and in the cases (p = 0.036).

**Table 1:** Detailed baseline characteristics of the study population for the HHPCP and CSPPT cohort

Variables	Controls (n = 1391)	Cases (n = 1391)	p value	Controls (n = 114)	Cases (n = 114)	p value
Male, n (%)	779 (56.0)	776 (55.8)	0.939	56 (49.1)	56 (49.1)	1.000
Age, y	69.35 (7.78)	69.35 (7.78)	0.998	60.85 (7.14)	60.86 (7.12)	0.988
BMI, kg/m^2^	25.57 (23.19, 28.07)	25.46 (23.12, 28.07)	0.525	25.23 (23.31, 27.46)	24.72 (22.76, 27.24)	0.231
Family history of cancer	47 (3.4)	50 (3.6)	0.836	0 (0.0)	1 (0.9)	1.000
Baseline SBP, mmHg	147.33 (133.83, 160.33)	146.00 (131.67, 161.00)	0.314	162.33 (154.67, 180.00)	162.00 (150.67, 172.50)	0.205
Baseline DBP, mmHg	83.00 (76.33, 90.33)	82.67 (75.00, 91.00)	0.249	96.67 (90.00, 101.83)	94.00 (86.50, 100.67)	0.281
History of diabetes, yes, no. (%)	137 (9.8)	191 (13.7)	0.002	7 (6.1)	3 (2.6)	0.332
Smoking status (%)			0.011			0.079
Current	334 (24.0)	401 (28.8)		33 (28.9)	49 (43.0)	
Former	160 (11.5)	163 (11.7)		8 (7.0)	5 (4.4)	
Never	897 (64.5)	827 (59.5)		73 (64.0)	60 (52.6)	
Alcohol consumption status (%)			0.359			0.735
Current	388 (27.9)	370 (26.6)		33 (28.9)	30 (26.3)	
Former	62 (4.5)	77 (5.5)		8 (7.0)	11 (9.6)	
Never	941 (67.6)	944 (67.9)		73 (64.0)	73 (64.0)	
TC, mg/dL	6.27 (5.38, 7.22)	6.36 (5.53, 7.23)	0.066	5.51 (4.81, 6.16)	5.51 (4.72, 6.16)	0.838
TG, mg/dL	1.14 (0.80, 1.75)	1.18 (0.81, 1.71)	0.409	1.42 (1.19, 1.98)	1.48 (1.17, 1.96)	0.691
HDL-C, mg/dL	1.16 (0.99, 1.36)	1.19 (1.03, 1.36)	0.033	1.19 (1.04, 1.47)	1.25 (1.04, 1.43)	0.785
Glucose, mmol/L	5.80 (5.30, 6.50)	5.80 (5.30, 6.50)	0.826	5.72 (5.22, 6.41)	5.46 (5.06, 6.12)	0.120
Homocysteine, µg/mL	12.01 (10.31, 14.66)	12.25 (10.06, 15.02)	0.904	13.00 (10.64, 17.00)	12.77 (10.17, 16.36)	0.389
Folate, ng/mL	6.14 (4.00, 9.65)	6.12 (4.23, 10.12)	0.437	6.80 (5.25, 9.76)	7.78 (4.98, 9.81)	0.601
MTHFR C677T			0.014			0.590
CC	339 (24.4)	288 (20.7)		26 (22.8)	24 (21.1)	
CT	832 (59.8)	837 (60.2)		59 (51.8)	54 (47.4)	
TT	220 (15.8)	266 (19.1)		29 (25.4)	36 (31.6)	
Betaine, µg/mL	5.73 (4.57, 7.11)	5.80 (4.58, 7.26)	0.401	9.38 (7.09, 11.83)	10.15 (8.30, 13.66)	0.036

Abbreviations: BMI: body mass index; SBP: systolic blood pressure; DBP: diastolic blood pressure; TC: total cholesterol; TG: triglycerides; HDL-C: high-density lipoprotein cholesterol; and MTHFR: methylenetetrahydrofolate reductase. Continuous variables are presented as median (quantile 1- quantile 3), categorical variables are presented as n (%). Differences in baseline characteristics between cases and controls were compared using chi^2^ tests for categorical variables and generalized paired t-tests and nonparametric Kruskal-Wallis tests for continuous variables

### Betaine and total cancer

**Fig. 2:**
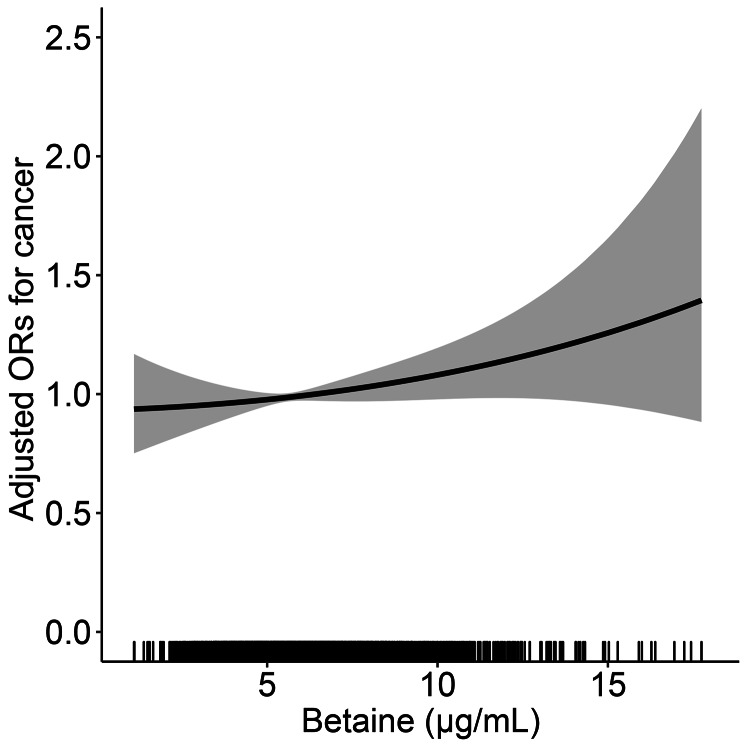
The association of betaine and the risk of total cancer using restricted cubic spline for the HHPCP cohort. Cubic spline graph of the adjusted OR (represented by the solid line) and 95%CI (represented by the dotted lines) **Notes**: Adjusted for age, body mass index, sex, smoking status, alcohol consumption, family history of cancer, diabetes, folate, homocysteine, systolic blood pressure, triglycerides, cholesterol, high-density lipoprotein cholesterol, glucose, and MTHFR C677T.

The relationship between serum betaine concentration and total cancer risk in the HHPCP cohort is shown in Fig. 2. There was a positive, dose-response relationship between serum betaine levels and cancer risk in the study participants; the risk of cancer increased as serum betaine increased. As shown in Table 2, there was a positive association between serum betaine as a continuous variable and total cancer (per SD increment, OR = 1.02, 95%CI = 0.99–1.06, p = 0.071) in the crude model. After adjusting for variables, the risk of total cancer increased by 3% for each SD increase of betaine (OR = 1.03, 95%CI = 0.99–1.07, p = 0.097). Unfortunately, there was no significant difference.

**Table 2:** The relationship between the concentration of betaine and the risk of total cancer and subtypes for the HHPCP cohort

Betaine (µg/mL)	Cases/controls	Crude model	p value	Adjusted model	p value
		OR (95%CI)		OR (95%CI)	
Total cancer	1391/1391	1.02(0.99,1.06)	0.071	1.03(0.99,1.07)	0.097
Lung cancer	361/361	1.04 (0.97,1.10)	0.261	1.03(0.96, 1.10)	0.361
Digestive cancer	544/544	1.05 (1.00,1.11)	0.073	1.07(1.01, 1.13)	0.025
Esophagus cancer	20/20	0.85 (0.69,1.06)	0.125	0.90 (0.67,1.22)	0.499
Gastric cancer	165/165	1.09 (0.99,1.20)	0.072	1.11 (0.99, 1.24)	0.068
Hepatic-biliary cancer	126/126	1.12 (1.00,1.26)	0.044	1.16 (1.02, 1.33)	0.021
Pancreatic cancer	52/52	0.90 (0.73,1.11)	0.319	0.85 (0.65, 1.11)	0.238
Colorectal cancer	181/181	1.05 (0.96,1.15)	0.323	1.08 (0.98, 1.19)	0.139
Breast cancer	86/86	0.98 (0.84,1.16)	0.840	1.00 (0.83,1.17)	0.886
Gynecologic cancer	47/47	0.95 (0.74,1.22)	0.674	0.99 (0.75,1.32)	0.983
Genitourinary cancer	130/130	0.95 (0.74,1.22)	0.674	0.99 (0.75,1.32)	0.983
Prostate cancer	59/59	0.94 (0.78,1.15)	0.561	0.94 (0.75,1.17)	0.563
Bladder cancer	41/41	1.08 (0.89,1.31)	0.460	1.10 (0.88,1.39)	0.393
Kidney cancer	30/30	0.92 (0.71,1.19)	0.513	0.82 (0.57,1.17)	0.271
Other cancer	223/223	0.96 (0.86,1.06)	0.376	0.94 (0.85,1.05)	0.302

Abbreviations: OR, odds ratios; CI, confidence intervals. Adjusted for age, BMI, sex, smoking, alcohol consumption, family history of cancer, diabetes, folate, homocysteine, systolic blood pressure, triglycerides, cholesterol, high-density lipoprotein cholesterol, glucose, and MTHFR C677T.

### The relationship between serum betaine and the risk of cancer subtypes

**Fig. 3:**
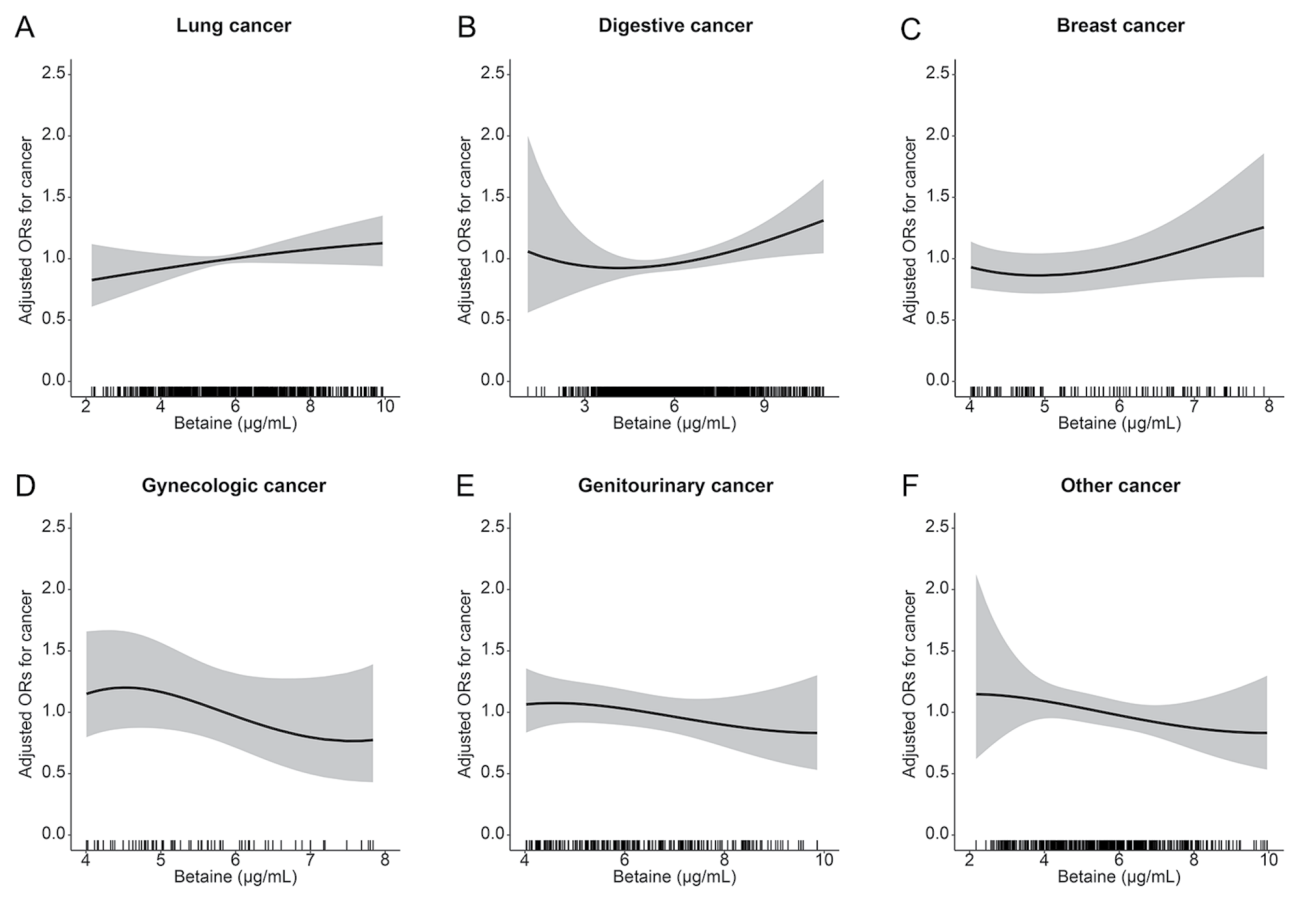
The relationship between serum betaine and the risk of cancer subtypes **Notes**: A, Lung cancer; B, Digestive cancer; C, Breast cancer; D, Gynecologic cancer; E, Genitourinary cancer; E, Genitourinary cancer; F, Other cancer; Adjusted for age, body mass index, sex, smoking status, alcohol consumption, family history of cancer, diabetes, folate, homocysteine, systolic blood pressure, triglycerides, cholesterol, high-density lipoprotein cholesterol, glucose, and MTHFR C677T.

Subgroup analyses for different tumor types were performed in the HHCPC cohort. The same pattern was observed in both the lung cancer subtype and the digestive cancer subtype. For lung cancer, serum betaine was positively associated with the risk of cancer (Fig. 3A). Interestingly, serum betaine had a U-shaped association with the incidence of digestive cancers (Fig. 3B). Overall, serum betaine was significantly, positively associated with digestive cancer risk (OR = 1.07, 95%CI = 1.01–1.13, p = 0.025), however, a turning point of 5.01 ug/mL yielded the best fitting model in a piecewise regression. When the level of serum betaine was less than 5.01 ug/mL, the risk of digestive cancer gradually decreased with the increase of serum betaine (OR = 0.82, 95%CI = 0.59–1.14, p = 0.228), and when serum betaine levels were equal to or exceeded 5.01 ug/ml, the risk of digestive cancer increased rapidly (OR = 1.08, 95%CI = 1.01–1.17, P = 0.036) (Table 3).

**Table 3:** The relationship between the concentration of betaine and the risk of digestive cancer in the HHCPC cohort

Betaine (µg/mL)	Cases/controls	Crude model	p value	Adjusted model	p value
		OR (95%CI)		OR (95%CI)	
Betaine < 5.01					
Continuous	158/176	0.84 (0.62,1.13)	0.243	0.82(0.59,1.14)	0.228
Quartiles					
Quartile 1 (< 3.68)	44/39	Reference		Reference	
Quartile 2 (3.68–4.25)	40/45	0.85 (0.46,1.55)	0.588	0.81 (0.43,1.56)	0.538
Quartile 3 (4.25–4.63)	40/44	0.81 (0.44,1.48)	0.486	0.69 (0.35,1.35)	0.281
Quartile 4 (4.63–5.01)	34/48	0.65 (0.35,1.19)	0.165	0.68 (0.35,1.33)	0.261
P for trend			0.578		
Betaine ≥ 5.01					
Continuous	386/371	1.06 (0.99,1.14)	0.088	1.08(1.01,1.17)	0.036
Quartiles					
Quartile 1 (5.01–5.86)	84/105	Reference		Reference	
Quartile 2 (5.86–6.73)	105/85	1.58 (1.05,2.37)	0.028	1.57 (1.03,2.40)	0.035
Quartile 3 (6.73–8.10)	98/91	1.36 (0.91,2.04)	0.137	1.48 (0.97,2.25)	0.071
Quartile 4 (≥ 8.10)	99/90	1.39 (0.93,2.08)	0.112	1.51 (0.98,2.33)	0.062
P for trend			0.152		

Abbreviations: OR, odds ratios; CI, confidence intervals. Adjusted for age, BMI, sex, smoking, alcohol consumption, family history of cancer, diabetes, folate, homocysteine, systolic blood pressure, triglycerides, cholesterol, high-density lipoprotein cholesterol, glucose, and MTHFR C677T.

Serum betaine had a positive association with the incidence of breast cancer (Fig. 3C). In gynecologic, genitourinary, and other cancers, an inverse association between serum betaine and cancer risk was observed (Fig. 3D-F). However, these association was not statistically significant. We further analyzed the relationship between the concentration of betaine and the risk of subtypes of digestive cancer. We found that serum betaine was negatively correlated with the risk of esophagus cancer and pancreatic cancer (the difference was not statistically significant), and positively correlated with the risk of gastric cancer and colorectal cancer (the difference was not statistically significant). There was a significant positive association between serum betaine and hepatic-biliary cancer risk (OR = 1.16, 95%CI = 1.02–1.33, p = 0.021) (Fig. S1 and Table 2).

**Fig. 4:**
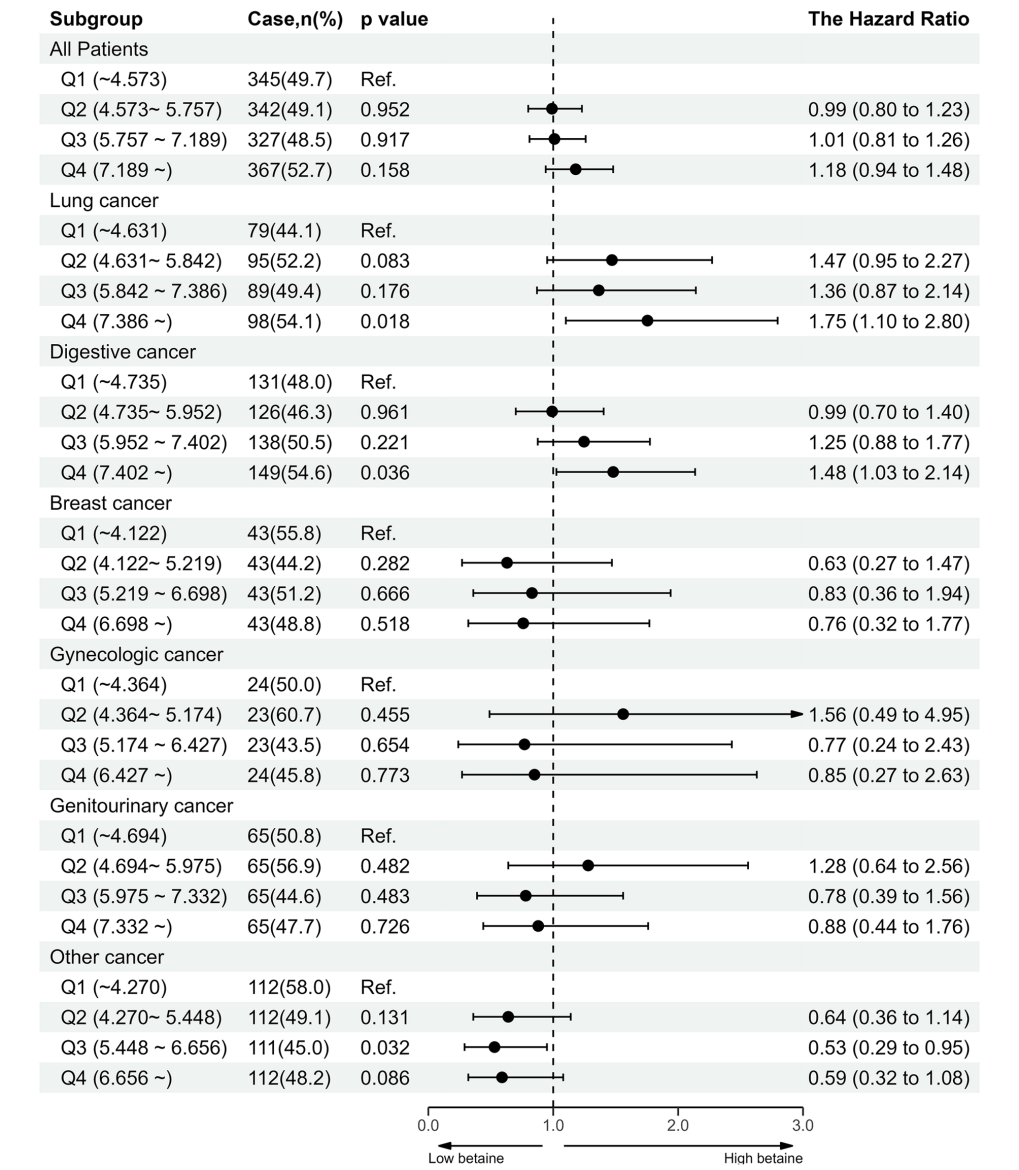
Forest plot displaying odds ratios associated with cancer subtypes by quartiles of betaine for the HHCPC cohort **Notes**: Adjusted for age, body mass index, sex, smoking status, alcohol consumption, family history of cancer, diabetes, folate, homocysteine, systolic blood pressure, triglycerides, cholesterol, high-density lipoprotein cholesterol, glucose, and MTHFR C677T.

Notably, a forest plot reveled that those participants with high serum betaine exhibited an increased risk of developing total cancer, lung cancer, digestive, and breast cancers, especially for those with high levels of betaine (Q4). For other cancers, serum betaine was inversely associated with the risk of cancer (Fig. 4). Subgroup analyses were performed to assess the effect of serum betaine on the outcome in various subgroups. A significantly stronger association between betaine and the risk of total cancer was found for younger participants (age < 65y), females, those with low folate (< 7.19 ng/mL), MTHFR rs1801133 (CT), current alcohol consumption, high total cholesterol (≥ 5.54 mmol/L), low triglycerides (< 1.69 mmol/L), and high high-density lipoprotein cholesterol (≥ 1.04 mmol/L). Notably, serum betaine had a significant interaction with high-density lipoprotein cholesterol. A significantly positive association between betaine and the risk of total cancer was presented in the high high-density lipoprotein cholesterol group (Fig. S2).

### External cohort validation of the relationship between serum betaine levels and cancer risk

**Fig. 5:**
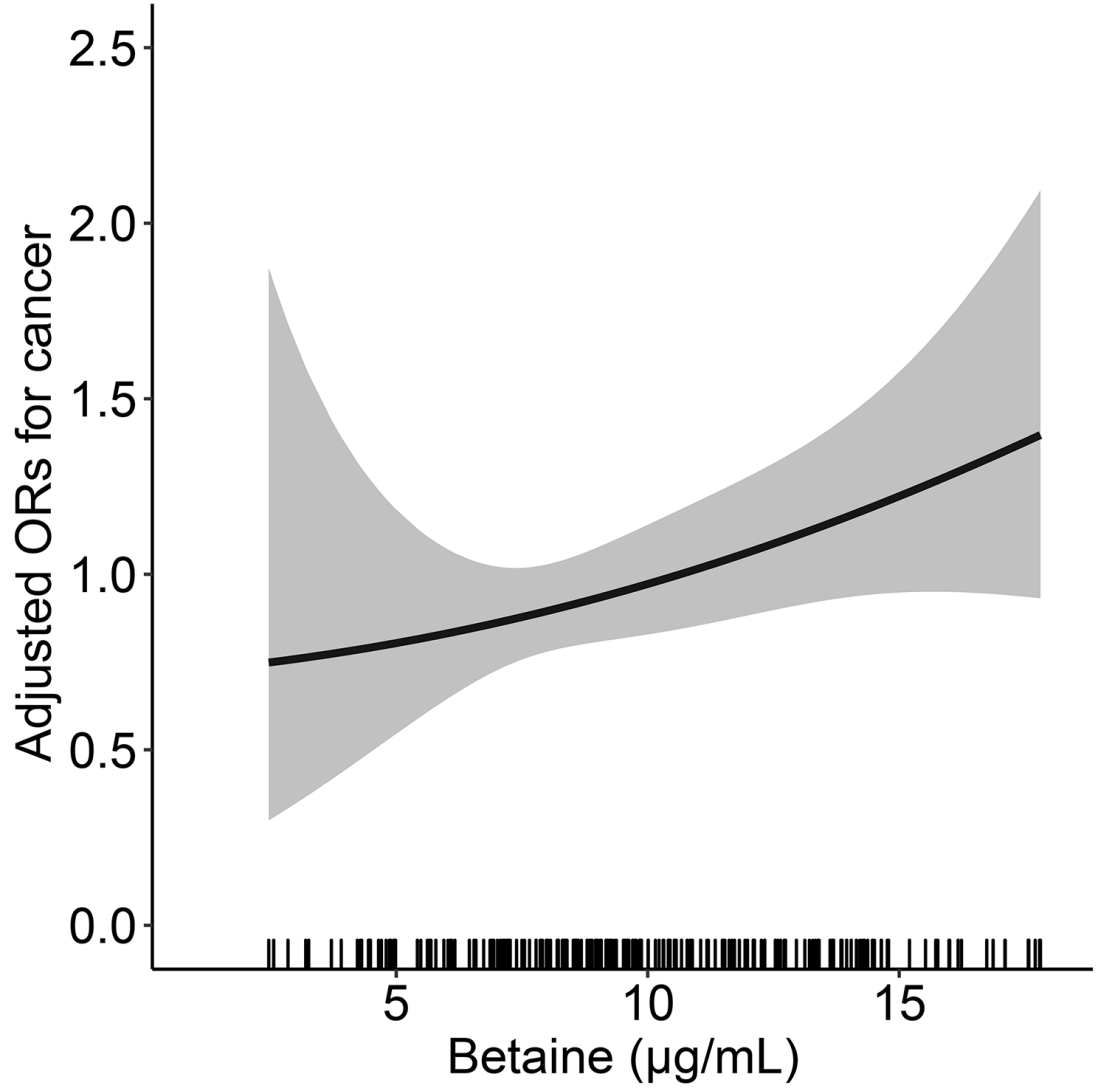
The association of betaine and the risk of total cancer for the CSPPT cohort **Notes**: Adjusted for age, body mass index, sex, smoking status, alcohol consumption, family history of cancer, diabetes, folate, homocysteine, systolic blood pressure, triglycerides, cholesterol, high-density lipoprotein cholesterol, glucose, and MTHFR C677T

The results of the multivariate-adjusted RCS analysis demonstrated a positive dose-response relationship between serum betaine and cancer risk in the CSPPT cohort (Fig. 5). There was a significant, positive association between serum betaine as a continuous variable and total cancer (OR = 1.34, 95%CI = 1.02–1.76, p = 0.038) in the crude model, and in the adjusted model (OR = 1.48, 95%CI = 1.06–2.05, p = 0.020). When serum betaine was modeled as quartiles, a significantly increased risk of cancer was found for those in quartile 4 (OR, 2.42; 95%CI, 1.06–5.53, p = 0.035) compared with those in quartile 1 (Table 4). Subgroup analyses revealed a positive correlation between serum betaine levels and the occurrence of lung, digestive, and other cancers (Fig. S3 and Table S1).

**Table 4:** The relationship between the concentration of betaine and the risk of digestive cancer for the CSPPT cohort

Betaine, µg/ml	Cases/Controls	Crude Model	p value	Adjusted Model	p value
		OR (95%CI)		OR (95%CI)	
Continuous	114/114	1.34(1.02,1.76)	0.038	1.48(1.06,2.05)	0.020
Quartiles					
Quartile 1 (< 7.63)	22/35	Reference		Reference	
Quartile 2 (7.63–9.66)	28/29	1.54 (0.73,3.23)	0.258	1.72 (0.75,3.94)	0.200
Quartile 3 (9.66–12.72)	30/27	1.77 (0.84,3.72)	0.134	1.58 (0.69,3.63)	0.281
Quartile 4 (≥ 12.72)	34/23	2.35 (1.11,4.98)	0.026	2.42 (1.06,5.53)	0.035
P for trend			0.025		0.042

Abbreviations: OR, odds ratios; CI, confidence intervals. Adjusted for age, BMI, sex, smoking, alcohol consumption, family history of cancer, diabetes, folate, homocysteine, systolic blood pressure, triglycerides, cholesterol, high-density lipoprotein cholesterol, glucose, and MTHFR C677T.

## Discussion

In this study, we demonstrated a positive association between high serum betaine and incidence of new-onset total cancers utilizing the HHPCP cohort and further validated this association in an external cohort (the CSPPT cohort). Among cancer subtypes, we found a positive association between serum betaine and the risk of lung cancer, and an inverse association with other cancers. Interestingly, we observed a U-shaped association between serum betaine and digestive cancers, with a turning point of 5.01 mmol/L for betaine. In addition, we found significant heterogeneity between serum betaine and the risk of digestive cancer subtypes. Serum betaine was positively associated with the risk of gastric, hepatic biliary and colorectal cancers, and negatively associated with esophagus and pancreatic cancers.

Butler et al. [23] found that serum betaine was associated with the risk of hepatocellular carcinoma, with the highest risk in the betaine > 75.8 µmol/L (about 8.9 µg/mL) group. De Vogel et al. found that although serum betaine was not significantly associated with prostate cancer risk, the OR of prostate cancer risk increased with serum betaine [21]. It is worth noting that Huang et al. [11] found that the relationship between serum betaine and the risk of pancreatic cancer was not linear but showed a trend of first decreasing and then increasing, which is consistent with our study. A recent study also found that baseline serum levels of one-carbon methyl donors and adrenergic compounds resulting from human and gut microbiota–mediated metabolism are associated with increased lethal prostate cancer risk [19]. However, there are also many studies with the opposite association. A 2014 case-control study found that serum betaine was significantly, inversely associated with colon cancer incidence [20]. Myte et al. [10] found that high serum betaine concentrations were associated with a reduced risk of colorectal cancer. A recent study of breast cancer also showed that serum betaine was significantly, inversely associated with breast cancer risk [9]. In addition, many studies have shown that dietary betaine intake can reduce the risk of breast, liver, and colon cancer [26, 27].

Inconsistencies in serum betaine levels among different studies may have contributed to these inconsistent conclusions. We found that in studies where serum betaine was a protective factor for cancer, serum betaine concentrations generally fluctuated at low levels (around 3.80 µg/mL). Far from reaching the threshold of increased cancer risk, these low levels may be the reason that no further observations of the relationship between high betaine levels and cancer risk were seen. The relatively higher betaine levels in our study population may have provided a possibility for us to observe the relationship between the upper limit of serum betaine and cancer risk. A recent study by Reichard et al. [19] found that serum betaine was associated with an increased risk of lethal prostate cancer. In this study, the serum betaine level of the cancer population fluctuated between 4.37 and 6.41 µg/mL, which can be considered a relatively higher level. In the HHPCP cohort, the serum betaine level of the cancer population fluctuated between 4.58 µg/mL -7.26 µg/mL, and we found a dose-dependent, U-shaped relationship between serum betaine and the risk of digestive cancers. In the CSPPT cohort, the serum betaine level was higher in the cancer population (8.3 µg/mL ~ 13.7 µg/mL), and there was a significant positive correlation between serum betaine and cancer risk.

The mechanisms underlying the apparent association between high concentrations of betaine and cancer risk are unclear, but we offer explanations it from the following aspects. It has been shown that a Western diet (one rich in energy, red meat, high-fat dairy, and processed foods) is associated with an increased risk of cancer [14–16]. One of the metabolic pathways of the Western diet is the production of TMAO through the gut microbiota-mediated metabolism of betaine compounds, choline, and carnitine. Therefore, in addition to providing methyl groups for DNA methylation, betaine can be metabolized to TMAO through another pathway. TMAO has been shown to be associated with an increased risk of various cancers [17, 18, 20]. Some studies have also shown that high concentrations of choline, a precursor of betaine, are associated with an increased risk of cancer [20, 22]. An elevated level of betaine may indirectly reflect the abnormal metabolism of serum choline. In addition, according to a previous study, the detrimental effect of both a deficiency and an excess of serum betaine might be caused by hyper- and hypo-methylation of DNA and proteins [28]. The relationship between serum betaine and cancer risk remains inconsistent. Our study found that high serum betaine may be associated with an increased risk of cancer, which provides new evidence for the relationship between betaine and cancer risk, and provides useful hints for monitoring dietary element intake, but more studies are still needed to further confirm our findings. It is our hope that this study might provide some evidence for future exploration.

The main strengths of this study are its nested, case-control design and the use of two independent cohorts, thus reducing selection bias and enhancing the credibility of the study. However, the limitations of this study should also be noted. First, this study did not record any detailed dietary information on betaine intake. Second, the participants in this study were all hypertensive patients, and whether our conclusions can be extrapolated to a population without hypertension or to a non-Chinese population remains inconclusive. In addition, the current mechanism of the relationship between serum betaine and cancer needs further exploration in future studies. Finally, due to the smaller number of cancer cases in the validation cohort, we were unable to test all the associations that were investigated in the discovery cohort. Therefore, more large-scale, and prospective studies are still needed to validate the results of this research.

## Conclusion

This study found that high serum betaine was associated with an increased risk of cancer. In terms of tumor types, serum betaine had a positive correlation with lung cancer, a U-shaped correlation with gastrointestinal cancer, and a negative correlation with other tumors. These analyses may have potential scientific and clinical implications for cancer prevention and dietary guidance. More research is still needed to confirm these findings.

### Electronic supplementary material

Below is the link to the electronic supplementary material.

Supplementary Material 1: Figure S1–S3 and Table S1

## Data Availability

Data described in the manuscript, code book, and analytic code will be made available upon request pending application and approval. Data will be made available upon reasonable request.

## Abbreviations

OR: Odds ratios
1C: One-carbon
TMAO: Trimethylamine N-oxide
HHPCP: China H-type Hypertension Prevention and Control Public Service Project
CSPPT: China Stroke Primary Prevention Trial
CI: Confidence interval
SBP: Systolic blood pressure
DBP: Diastolic blood pressure
RCS: Restricted cubic spline regression
