# Supplementary material for: The association of serum betaine concentrations with the risk of new-onset cancers: results from two independent nested case-control studies
Source: Nutr Metab (Lond). 2023 Oct 30;20:46. doi: 10.1186/s12986-023-00755-y (PMC10614375; doi:10.1186/s12986-023-00755-y)
Supplement: Supplementary file 1 — Supplementary Material 1: Figure S1–S3 and Table S1 [file 12986_2023_755_MOESM1_ESM.docx]

**Supplementary Figure 1**. The association of betaine and the risk of subtypes of digestive cancer using restricted cubic spline for the HHPCP cohort.

**
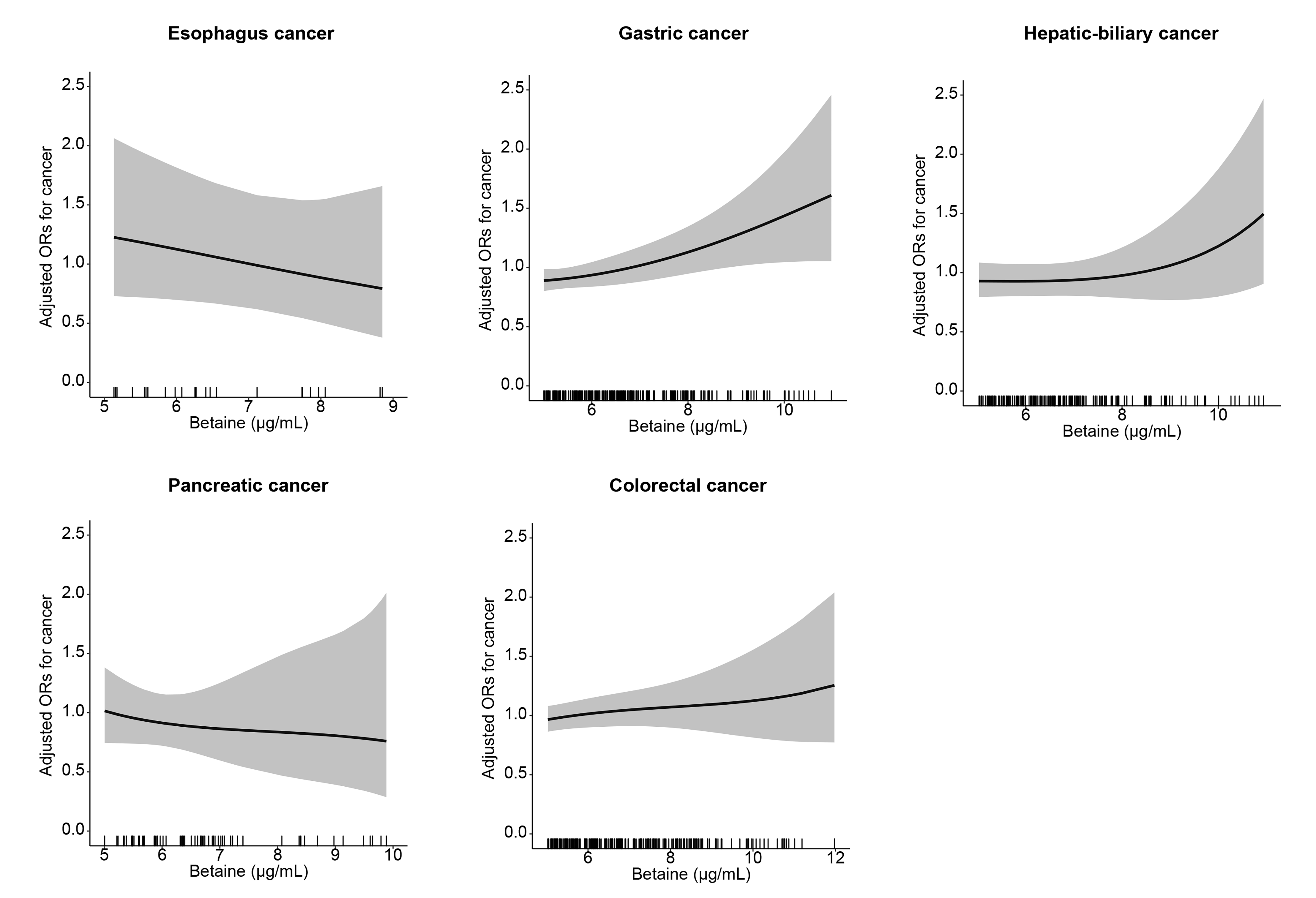
**

Notes: Adjusted for age, BMI, sex, smoking, alcohol consumption, family history of cancer, diabetes, folate, homocysteine, systolic blood pressure, triglycerides, cholesterol, high-density lipoprotein cholesterol, glucose, and MTHFR C677T.

**Supplementary Figure 2**. Forest plot displaying odds ratios associated with total cancer by quartiles of betaine for the HHCPC cohort.


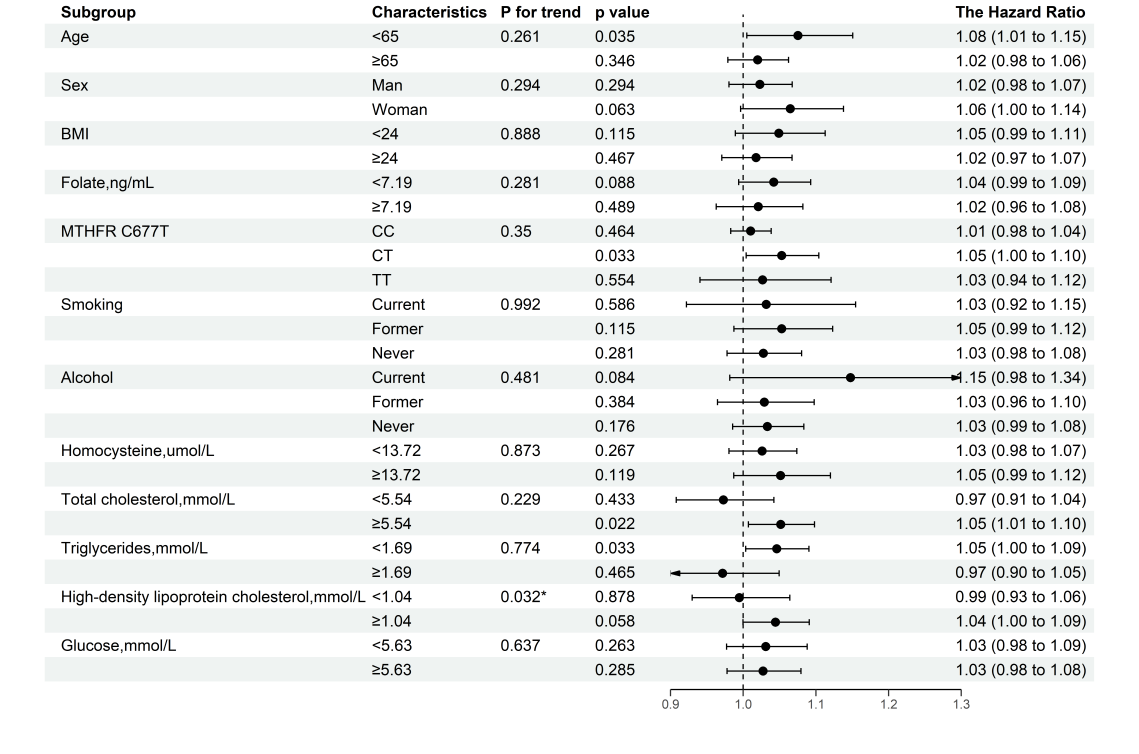


**Notes:** Adjusted for age, body mass index, sex, smoking status, alcohol consumption, family history of cancer, diabetes, folate, homocysteine, systolic blood pressure, triglycerides, cholesterol, high-density lipoprotein cholesterol, glucose, and MTHFR C677T.

**Supplementary Figure 3**. The association of betaine and the risk of subtypes of cancer using restricted cubic spline for the CSPPT cohort.


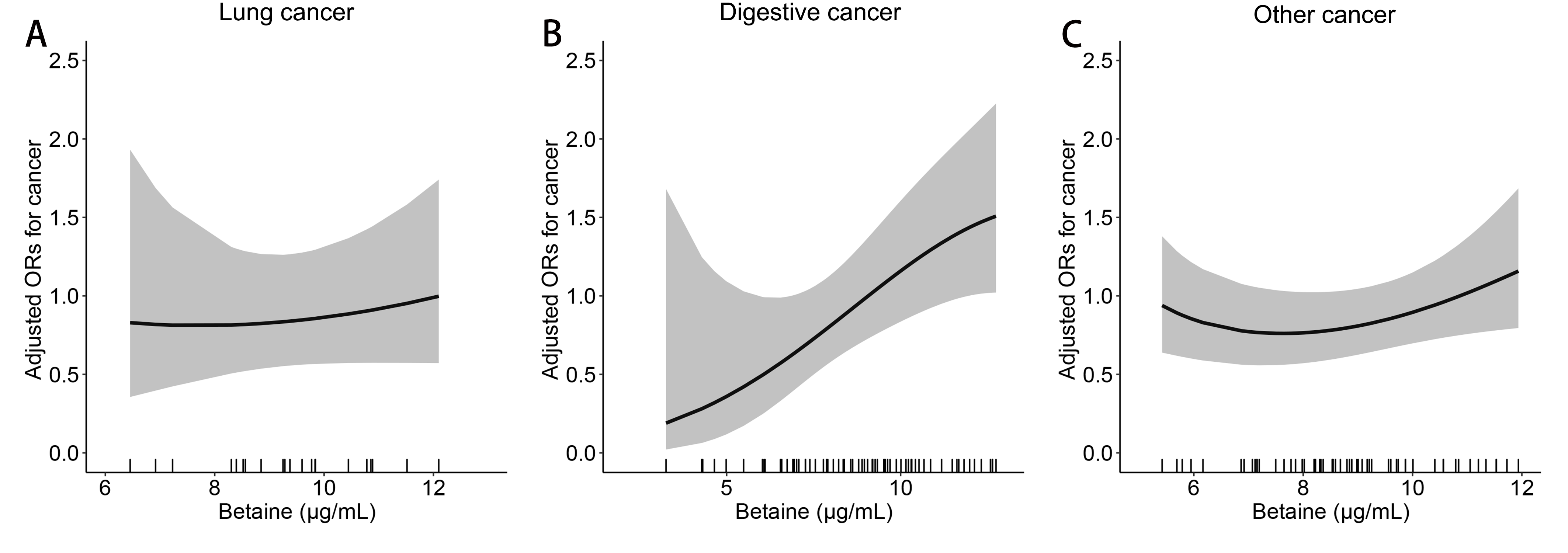


**Notes:** A, Lung cancer; B, Digestive cancer; C, Other cancer;

Adjusted for age, body mass index, sex, smoking status, alcohol consumption, family history of cancer, diabetes, folate, homocysteine, systolic blood pressure, triglycerides, cholesterol, high-density lipoprotein cholesterol, glucose, and MTHFR C677T.

**Table S1.** The relationship between the concentration of betaine and the risk of cancer subtypes for the CSPPT cohort.

| **Betaine (µg/mL)** | **Cases/controls** | **Crude model** | **p value** | **Adjusted model** | **p value** |
| --- | --- | --- | --- | --- | --- |
|  |  | **OR (95%CI)** |  | **OR (95%CI)** |  |
| Lung cancer | 17/17 | 1.07 (0.94,1.21) | 0.297 | 0.98(0.82, 1.17) | 0.823 |
| Digestive cancer | 49/49 | 1.11 (1.00,1.23) | 0.040 | 1.10(0.98, 1.24) | 0.118 |
| Other cancer | 48/48 | 1.03 (0.93,1.15) | 0.594 | 1.04(0.93, 1.16) | 0.512 |

Abbreviations: OR, odds ratios; CI, confidence intervals. Adjusted for age, BMI, sex, smoking, alcohol consumption, family history of cancer, diabetes, folate, homocysteine, systolic blood pressure, triglycerides, cholesterol, high-density lipoprotein cholesterol, glucose, and MTHFR C677T.
